# COVID‐19 and democratic resilience

**DOI:** 10.1111/1758-5899.13137

**Published:** 2022-09-01

**Authors:** Richard Youngs

**Affiliations:** ^1^ Carnegie Europe and University of Warwick – Politics and International Studies Coventry UK

## Abstract

The COVID‐19 experience has sharpened debates about democracy's future worldwide. In reflecting on these debates, this paper does three things. First, it assesses how resilient democracy has been in the COVID‐19 emergency. Second, it examines the effect the pandemic has had on the pre‐existing trends in democratic politics. And third, it suggests ways in which the COVID‐19 crisis both requires and possibly opens the door to democratic rejuvenation.

## 1 INTRODUCTION

In the years preceding the COVID‐19 crisis, democracy was in fragile health. Throughout the 2010s, academic analysis focused heavily on democracy's problems and its apparent unravelling in many parts of the world. Since the beginning of 2020, the pandemic has placed additional strains on democratic institutions and practices. It has also intensified efforts at many levels to improve and defend democracy. The COVID‐19 experience has sharpened debates about democracy's future worldwide.

In reflecting on these debates, this article does three things. First, it assesses how resilient democracy has been in the COVID‐19 emergency. Second, it examines the effect the pandemic has had on the pre‐existing trends in democratic politics. And third, it suggests ways in which the COVID‐19 crisis both requires and possibly opens the door to democratic rejuvenation. The article was prepared in the context of a Club de Madrid policy dialogue; as such, it does not represent a comprehensive academic study of these issues, but rather extracts a selection of political lessons from the COVID‐19 pandemic.

## 2 HOW RESILIENT HAS DEMOCRACY BEEN?

Analytical and policy debates have increasingly delved into the complex question of what can be learned from different political responses to COVID‐19. Democracy has proven resilient in some countries, less so in others – resilience being understood as political systems' ability to absorb and weather the effects of crisis and recover from them. Analytical attention has turned to drawing lessons from well‐performing democracies and understanding what has helped make both their formal institutions and wider democratic culture resilient. And conversely, it faces the challenge of isolating the precise reasons for shortfalls in democratic resilience in other cases.

Quantitative studies show that the level of democracy worldwide has declined in significant ways during the pandemic. According to the Economic Intelligence Unit, in the pandemic's first year of 2020 over half the countries in the world saw declines in their democracy scores. The situation got even worse in 2021. The global democracy score fell from 5.37 to 5.28, a sharper decline even than in 2020 and the lowest score since the index started in 2006. The pandemic resulted in a narrowing of civil liberties among developed democracies and authoritarian regimes alike. The decline in civil liberties 2019–21 occurred in all regions and accelerated notably compared to the preceding years. The imposition of vaccine passes raised difficult questions about individual freedoms. Even if many developments were not directly caused by the pandemic, there were major violations of rights norms in 44 countries due in some measure to the pandemic and moderate violations in 57, while COVID‐19 also reduced levels of mass mobilization overall for both 2020 and 2021 (Economist Intelligence Unit, 2021).

Lying behind COVID‐19's menace to democratic resilience, three quite distinct dynamics have been evident. One is that of governments using restrictive legal or emergency measures in their genuine attempts to mitigate the COVID‐19 emergency, laying aside democratic rights within the limits of the law. Another is that of governments using emergency measures in disingenuous fashion to tighten their own hold on power and hollow out democratic checks‐and balances. And finally, is the wider challenge of COVID‐19's ravages rendering more difficult the effective exercise of democratic rights and functioning of formal democratic institutions. As the cycle of pandemic has unfolded, the main challenge has shifted from the first to the second and then to the third of these dynamics (Youngs & Panchulidze, 2020).

The second dynamic of disingenuous executive aggrandizement (Bermeo, 2016) has been especially notable in countries that were already undergoing democratic erosion or autocratic empowerment. While many democracies have suffered, some authoritarian states have emerged with stronger means of control due to the pandemic. Governments in Cambodia, the Philippines, Singapore and Vietnam among many others arrested hundreds of people alleging their criticisms of COVID‐19 management to be fake news. In Egypt, many doctors were arrested during the pandemic for publicly criticizing the government's coronavirus response. In Turkey, state repression increased against journalists after news reports challenged official coronavirus figures, with journalists often charged with provoking the public and inciting public fear and panic. In Algeria, authorities used the pandemic to suppress the opposition and end the Hirak protest movement.

In Russia, activists were targeted for drawing public attention to the government's inadequate supplies of medical equipment. In Azerbaijan, the government took advantage of the opportunity to arrest opposition politicians for alleged quarantine violations. The Polish government tightened restrictions on civil liberties, dismissed healthcare workers who had spoken out about bad conditions in their institutions, and sought to limit civil society organisations' (CSOs') access to foreign funds. In Serbia, the government self‐servingly lifted restrictions before an election, tightening its grip on power. And in Zimbabwe, the deployment of soldiers to conduct policing duties in townships helped the regime's autocratic consolidation, as they detained many opposition figures under COVID pretexts (for more on all these examples, see Youngs et al., 2021).

Most strikingly, China has become even more repressive and restrictive of rights, as it moved to head off societal unrest. One analytical line is that China's strong containment of COVID‐19 shows the need to sacrifice liberal freedoms for the sake of good public outcomes (Li, 2021). At the international level, a widespread concern is that some democracies' clear mismanagement of the crisis has left a dent in global dynamics supportive of democratic norms. The fact that China has provided far more vaccines to developing states than Western countries may risk undermining democracy's global appeal.

As a counterbalance to these trends, other evidence points towards a degree of democratic resilience. While the pandemic's early stages saw widespread concern that the negative impact on liberal politics could be overwhelming, as the crisis has unfolded the effect on democracy has perhaps not been as dramatic across the board as many initially feared might be the case. In the Omicron wave of early 2022, in general governments were less sweeping in re‐introducing restrictions than in earlier waves of the pandemic. While countries that were already suffering major democratic erosion have seen that negative trend deepen, in states with higher quality democracy the effects have been more subtle.

Data from the Varieties of Democracy institute show this differentiated pattern. By July 2021, most democracies had recorded minor or moderate violations. Of the 30 countries experiencing only minor infringements, 22 were democracies. Of the 44 states that suffered major violations, 30 were autocracies. Overall, non‐democratic or partially democratic states experienced more serious infringements in political rights. The situation has improved over time: for the second quarter of 2021, 83 states saw no or only minor violations, compared to 48 for the same period in 2020 (Gothenberg, V‐Dem Insitute, 2021). The EUI Index's biggest declines for 2021 – Afghanistan, Myanmar, Tunisia – were not COVID related.

On the positive side of the equation, resilience has come from different sources. Some academic attention has started turning to comparative assessment and quantitative measurement of democratic resilience (GMF, 2021; Luhrmann & Merkel, 2021). In many democracies, courts and parliaments have pushed back against executives' use of emergency measures and watered them down. In around a dozen EU states, governments were pushed into introducing measures to boost judges' independence in 2021. In some cases, courts even forced governments to retract measures of genuine utility in fighting the virus, like some quarantine and testing rules. Surveys suggest that citizens' distrust in democratic governments has evolved in very different ways across different countries due to COVID‐19, with not all countries seeing a deterioration (Edelmann, 2021).

In some ways, citizens' scepticism has kept a healthy critical pressure on governments and helped improve authorities' COVID‐19 strategies. Democratic protests and activism have proven robust since early 2020; indeed, protest intensity has actually increased in many states and has in many cases focused on calls for democratic reform. The COVID‐19 pandemic has in some countries added to the demands of protestors and intensified pressure for democratic change.^1^

In addition to protest, COVID‐19 created an opportunity for greater democratic innovation by governments and citizens working together. Most major cities, for example, have either widened existing or created new participative platforms to engage local populations in COVID‐19 recovery plans (Youngs, 2021). Other kinds of institutional resilience have been evident too. South Korea's 2020 elections serve as a best‐practice model and garnered the country's highest turnout since 1992. New Zealand held an enquiry into crisis management that was headed by the opposition and served to pre‐empt political polarization. A new hotline in India was set up to report over‐priced medical equipment. Even amid largely negative developments for open politics, the pandemic also spurred many layers and forms of putative democracy‐deepening.

## 3 IMPACTS ON BROADER DEMOCRATIC EROSION

A crucial issue is how these COVID‐19‐related developments interact with the broader trends in global democracy that predated the pandemic. In the years before COVID‐19, scholars of democracy were concerned principally with charting and explaining a gathering global trend of democratic regression. Academic work of this era focused overwhelmingly on uncovering the factors behind de‐democratization, democratic erosion and authoritarian resurgence. Writers suggested a range of explanatory factors to this end. At the global level, as non‐democratic powers gained influence, the international system ceased to provide a favourable context for democratic change. Authoritarian regimes seemed to learn more effective tactics to undercut democratic processes. A core debate was over the relative weights of economic and identity‐related factors in explaining democracy's malaise. Many theorists stressed digital technology as an epoch‐defining factor corroding democratic debate and agency. Different writers placed their emphasis on different factors, but in general concurred that the range of drivers at work was extensive and powerful in prompting democratic decay (Berman, 2018; Diamond, 2019; Foa & Mounck, 2017; Keane, 2020; Levitsky & Ziblatt, 2018; Luhrman & Lindberg, 2019; Runciman, 2019; Snyder, 2019).

Analysts have stressed how COVID‐19's political effects grafted themselves on to this ongoing malaise in global democracy, making it academically important to examine the relationship between the pandemic and these pre‐existing trends (Diamond, 2021). As many countries move towards a recovery phase, it is instructive to consider precisely what kinds of additional strains COVID‐19 has placed on democracy and where it has added particularly acute risks to democratic resilience – and conversely where it has perhaps begun to mitigate some of these wider strains.

While COVID‐19 has dominated much political debate since early 2020, it is only one factor among many that are reshaping democracy worldwide. Democracy stands challenged in significant ways well beyond the pandemic's impact – and democratic institutions must be prepared for other kinds of crisis and emergency likely to emerge in the future. Prominent theorists suggest that democracy has shown itself to be an always‐evolving set of practices, rather than a static concept in need of preserving from imminent redundancy (Muller, 2021). COVID‐19 factors and other political dynamics have increasingly intertwined with each other in complex ways. While a comprehensive academic survey of all these effects is well beyond the scope of this article, the Club de Madrid policy dialogues that informed this article identified a select number of issues deemed to be of particular concern. These issues are highlighted here as those where there seems to be a particularly striking and mutually reinforcing *intersection* between COVID‐19 impacts and democracy problems that were accumulating before the pandemic began.

### 3.1 Governability

Concerns have been mounting for years over democracies' ability to provide effective governance (Doorenspleet, 2018). This has now become an even more important source of concerns about institutional resilience. If democracy has appeared shaky in many places since 2020, it has been in some measure because governments' basic decision‐making effectiveness has left much to be desired. Decision‐making efficiency and responsiveness worsened. Poor governance has sapped crisis management, and in turn, COVID‐19 has itself intensified these same problems of governability.

This is reflected at the level of both the state and political parties. The pandemic revealed even more clearly a shortcoming that was already becoming more consequential during the 2010s, namely the lack of state capacity to devise and implement public policy in ways that could more effectively underpin democracy and political rights (Fukuyama, 2020). Some authoritarian and hybrid regimes proved to be more effective in mobilizing state capacity than many democracies were in response to the pandemic (Brown, 2022). Additionally, in many countries, differences widened and political parties struggled to form stable governing coalitions. Parties seemed less willing or able to compromise around practical problem‐solving agendas. Empirical research suggested that COVID‐19 management had different impacts on polarization: the actions of highly partisan governments intensified political and social tensions, whereas in countries with broader based coalitions or expert‐led crisis management the pandemic served to dampen polarization (Flores et al., 2022). The pandemic has heightened the significance of these contrasting read‐overs between democratic quality and effective governance.

### 3.2 Civil liberties and civic space

Restrictions on civil society organizations are worsening in many democracies. This reflects a trend that has been present and gathering greater force for at least a decade. The constriction of civil society has become a global phenomenon driven by multiple factors (Chaudhry, 2022). Some newer restrictions are related to COVID‐19, or at least governments justify them by reference to the health emergency. Other restrictions reflect other issues or are justified in other terms, like security. Restrictions on civil liberties are the main source of the declining democracy scores of democratic states; this is a problem both driven by and far wider than COVID‐19.

Civicus's 2021 State of Civil Society Report shows deepening problems especially in authoritarian states but in democracies too. The Civicus Monitor, a participatory research platform that measures civic space globally, uncovers rising concerns over state surveillance, an increase in the coercive capacities of law enforcement agencies to enforce lockdowns and restrictions on human rights defenders and organizations uncovering grave human rights violations and high levels of corruption. Those seeking to protect the rights of the politically, economically or socially excluded, particularly in remote locations, have been most at risk (Civicus, 2021).^2^ Into 2022, in a large number of countries, these civic restrictions were still becoming more severe, even where COVID‐19 seemed to be abating somewhat and governments were generally loosening health controls. These restrictions were both direct and indirect: some were directly linked to the health situation, while others were related more to the much broader social and political spill‐over from the pandemic (Narsee, 2022).

### 3.3 Populism

Many populist leaders and parties have fared badly and lost support during COVID‐19 (Foa et al., 2022; Meyer, 2022), even if many retain significant levels of support and a few have even gained ground. Perhaps most significantly, populism may be shifting shape. In some democracies, populist‐style groups are fashioning an agenda around opposition to government intrusion and moving away somewhat from such an overwhelming focus on migration. In Europe, hard‐right populists shifted strategy to coordinate across borders around a critique of the EU using the pandemic to intervene undemocratically in member states' respective national politics (Lamour & Carls, 2022).

Different strains of populism seem to be forming: some illiberal leaders have used top‐down powers to speed up vaccines and bear down on new variants, whereas others have used the pandemic to advance a more libertarian agenda against state powers. In some regions like Latin America, populists may gain from a focus on COVID‐19's accentuation of inequalities, while in other regions like North America or Europe they tap into more of an anti‐state feeling. Another emerging populist focus with some apparent public resonance is on the need to ensure accountability over health experts as these gained in prominence during the pandemic (Maduro & Kahn, 2020). These diverse trends in populism have different implications for democracy and will require different kinds of counterstrategies.

### 3.4 Participation

For around two decades, many party memberships and electoral turnouts have suffered serious declines. At the same time, citizens have participated in higher numbers in other forms of democratic engagement. The pandemic reinforced the need for engagement by highlighting the importance of connections and trust in society: whether people are engaged with one another and with their institutions affects their willingness to wear masks, follow safe distancing practices and get vaccines. Many people sought out engagement with one another in order to retain their connections and help each other with day‐to‐day needs during the crisis. In parallel, mass protests about COVID‐9 issues have often morphed into efforts to build community level self‐organization. Not all such mobilizations are about democracy or even necessarily favourable for democratic reform, but a large number of them have been.

While most governments introduced restrictions on civil society activities as part of their initial crisis management strategies, the coronavirus pandemic story is not only about the closing of civic spaces but also about the opening of new civic spaces. These emergent civic spaces have, in some countries, involved national and local authorities facilitating CSO and social movement cooperation on health, social, economic, and community service provision issues. One of the most striking trends has been the spread of self‐organization aimed at practical problem solving related to the pandemic. Since the coronavirus first broke out in late 2019, many civic organizations have formed and organized themselves around practical types of community action. Academics allude to a more ‘agonistic’ form of democratic participation taking shape due to the pandemic (Suss, 2021).

More structured forms of deliberative participation, like citizen assemblies, have also gained support. Even as experts were called upon for their advice during the pandemic, COVID‐19 has given a further boost to both protest activity and organized participative experiments. In particular, the pandemic has inspired digital democratic innovations, as COVID‐19 disrupted existing patterns of engagement and forced public officials, their staff, and citizens to adapt to a world where face‐to‐face meetings were impossible. This has spurred newer democratic innovations to serve the pandemic's most pressing needs: generating verified information and reliable data; mobilizing resources, skills, and knowledge to address the health emergency; connecting volunteers and service organizations with people who needed help; and implementing and monitoring public policies and actions (Pogrebinschi, 2021).

### 3.5 Disinformation

Few issues have received more attention over the last decade than the rise of disinformation and a wider set of pernicious digital practices that distort basic principles like truthfulness and access to reliable information. The relationship between disinformation and democracy is complex. One risk lies in the way that disinformation itself makes it harder for citizens to exercise high quality democratic rights. But an equally real danger has arisen of governments' counter‐disinformation strategies also infringing core freedoms and rights. Many democratic governments have in recent years introduced laws to regulate online activity more strictly, taking aim at both external government‐sponsored influence operations and the social platforms. Yet many of the same governments have also used digital means to empower themselves, narrow democratic freedoms and increase surveillance.

COVID‐19 turbo‐charged the prevalence of disinformation and demonstrated even more clearly the damaging effects it can have. Political, medical and religious authorities teamed‐up for one counter‐disinformation initiative called the International Roundtable on Vaccination. Yet, counter‐disinformation was also a gift to non‐democratic governments. Many authoritarian regimes like Russia introduced new measures and fines against those spreading COVID‐19 disinformation that were used against political opponents.

### 3.6 International dynamics

International efforts to defend and extend democratic norms appear to have waned over the last decade. Some international cooperation has certainly taken place in defence of democratic breakthroughs, for example in Ukraine or in development‐aid commitments around the Social Development Goals. But, overall a more realpolitik tone has become apparent in international relations. Most democracies have increasingly given priority to their economic and security interests. Again, the international dimensions of COVID‐19 have accentuated this set of challenges. The pandemic seemed to intensify the malaise of the liberal international order and require more effort to defend this order as a foundation for democratic norms (Ikenberry, 2020).

Most states engaged in zero‐sum policies to protect their own interests and citizens, with even the established democracies often flouting global rules and norms to do so. China's provision of vaccines around the world and its assertive COVID‐19‐related diplomacy have added a further dimension to sharpened geopolitical rivalries across the world. Western governments' refusal to allow in those with Chinese vaccinations damages the image of the democratic world in the eyes of many citizens around the world. As COVID‐19's affects were becoming stronger, in mid‐2021 events in Afghanistan quite separately added further doubts to the future of the international democracy support agenda. It was striking in 2020 and 2021 how the national and international elements of the crisis seemed to fuse in shaking liberal norms and values.

## 4 PRIORITIES FOR DEMOCRATIC RENOVATION

Identifying these different thematic challenges highlights the factors that will do most to condition democracy's fate coming out of the COVID‐19 pandemic and reveals the sites of reform upon which the vitality and resilience of democratic institutions and practices will depend. These relate to the areas where the COVID‐19 experience has had a major impact on pre‐existing democracy agendas – either by aggravating already‐present challenges or opening new opportunities for positive change. Some of these issues flow directly out of the COVID‐19 experience, while other challenges have been more indirectly accentuated by the pandemic. A number of factors will be especially apposite to the question of whether democracy can be made more resilient and responsive.

### 4.1 Democratic crisis readiness

There are underlying reforms that would help democracies deal more effectively in the future with such serious events. The experience of the last 2 years suggests that democracies will need updated emergency measures that include better accountability mechanisms and will in the future allow effective government crisis responses but also with societal involvement and oversight.

For future crises to be tackled in more democratic ways channels of civic involvement will need to be developed and harnessed to ensure early‐warning signals find more timely response from state authorities. New emergency measures will need to guarantee better access to information in future crises as a means of underpinning the legitimacy and resilience of state institutions. They will need to ensure that minorities are more fully involved – as one example of why this is important, indigenous communities in Latin America and elsewhere have been largely excluded and this has complicated COVID‐19 management in 2020 and 2021. If global democracy is not to take another hard hit in future crises, responses will need to be more participative and also include groups that are more fully inclusive and representative of their societies.

### 4.2 Democratic civics

The pandemic has revealed the need for stronger and more dense networks of civic organization to underpin democratic resilience. The assault on civil society that was underway well before the pandemic makes this an especially deep‐rooted imperative: stronger and more widely cast local organization is essential to withstanding democratic governments' subtle and piecemeal narrowing of civil liberties and the democratic space for self‐organization. COVID‐19 revealed how important such active citizenship is in emergency situations to bolster government policies and formal institutions.

The rich networks of democratic innovation, deliberation and mobilization that have crystalized in recent years provide a dynamic launchpad: these will need to be extended to involve greater number of citizens and to ensure that protests, assemblies and organized CSO campaigns work together more smoothly with each other – both in normal times and emergency periods. All these actors will need to coordinate to ensure that all civic restrictions are removed fully, and that COVID‐19 is not used as a pretext for a more controlled form of democracy. Close attention will need to be given to monitoring what happens in the post‐COVID‐19 period, to ensure that the pandemic's legacy is not one of weaker civil society and stronger police powers. CSOs will need to lead this effort, and often call to account democratic governments for failing to comply with their own rhetoric.

### 4.3 Democracy and state capacity

Much debate in 2020 and 2021 has focused on how the pandemic shines a stronger spotlight on the need for stronger state capacities to reduce inequality not only in economic opportunity but also the exercise of political rights. It is widely recognized that the COVID‐19 experience has tipped the scales decisively in favour of more interventionist economic policy (Economist, 2022; Tooze, 2021). In COVID‐19's shadow, analysts have identified the presence or absence of central state‐government power as increasingly crucial in explaining the success or failure of democratization (Pildes, 2021). Yet if this shift in economic doctrine is to be beneficial and not detrimental to democratic quality, then the development of better state services will need to go hand in hand with wider civic involvement in deciding on these. The pandemic shows how democratic resilience requires a smoother dovetailing between formal institutions and informal citizen mobilization around efforts to reduce social and economic inequalities. A correct balance is needed between societal pluralism and state efficacy.

This kind of accountability and civic engagement are still relatively under‐developed in relation to governments' large recovery aid packages of support; debates will inevitably sharpen over where this money goes, and these decisions will need to be made in inclusive and participative ways if the funding is to help rebuild trust and legitimacy in government. The understandable desire to get such funds allocated quickly is already generating problems of corruption, as contracts fail to meet the highest standards of financial probity: international organizations and new democracy initiatives will need to pay particular attention to this challenge in the next several years. Cases like Brazil show how a lack of government transparency is undermining de facto democratic accountability over socio‐economic measures. Many of the same democratic governments that profess a commitment to defending democracy are also guilty of indulging corrupt practices both domestically and internationally.

### 4.4 Dealing with post‐COVID populism

Democracies will need to deal with the illiberal movements triggered by frustrations with COVID‐19 restrictions. These emergent movements are a part of civil society and can be seen as signs of a healthily critical citizenship but will need to be channelled into firmly democratic directions. Democratic reformers will need to adjust their counter‐populism strategies if and when these movements and parties gain traction from a ‘reigning in the executive’ narrative. It may be more difficult simply to demonize this variant of populism as anti‐democratic than with pervious variants of populism. There may be an opportunity for democratic‐liberals to build the case with some such groups that ‘protecting the individual from state intrusion’ actually requires the very checks and balances and constitutional guarantees towards which many populists have been somewhat cavalier in recent years.

### 4.5 International democratic coordination

Democratic resilience must also be boosted at the international level and cannot be ensured by national or sub‐national reform initiatives alone. In the face of Chinese and Russian vaccine diplomacy and a perception that some autocracies, like China, have handled the crisis well, sustained effort will be needed to develop a narrative on democracy's advantage in emergencies. The importance of better regional responses in the future to protect vulnerable democracies needs to be more fully recognized in this sense.

The US‐led Summit for Democracy in December 2012 was a promising move in this direction. Participant governments will need fully to implement the commitments they made at and after the summit to improve their own democracies and to strength their external support for democratic norms. In the run up to a second such summit currently scheduled for early 2023, civil society leaders will need to make this point forcefully in trying to widen the summit’s agenda into a fully comprehensive approach to democratic challenges. They will need to push to make sure this new international process connects with the other issues discussed in this report, like equality of rights and democratic state‐capacities.

Of course, at the time of this writing, the Russian invasion of Ukraine has dramatically increased the commitment to international coordination among democracies; while it is understandable that the invasion is now the primary driver of democracy‐protection, the challenges related more specifically to COVID‐19 still need to be addressed. The fragilities revealed and worsened by the pandemic should not be forgotten, even amid the tragedy of Ukraine's plight, if democratic resilience is to be rebuilt and enhanced around the world.

## Data Availability

Data sharing is not applicable to this article as no new data were created or analyzed in this study.

## Endnotes

^1^
For a running update on these protests, see Carnegie's Protest Tracker: https://carnegieendowment.org/publications/interactive/protest‐tracker?gclid=CjwKCAjwtcCVBhA0EiwAT1fY7yUB4fZUiMFtzO3MNpc03BVR9BPDQk8PkpyMvSkuhJnASXUNoGdI5BoCX3UQAvD_BwE

^2^

https://civicus.org/state‐of‐civil‐society‐report‐2021/ https://monitor.civicus.org/COVID19May2021/
